# Morphometric Evaluation of Various Segments of Adult Human Dry Humeri and Their Clinical Applications

**DOI:** 10.7759/cureus.115569

**Published:** 2026-08-31

**Authors:** Abishek Robinson Vasudevan, Chitra Nagaraj, Shyamala Bhadravathi Yadurappa, Seema Shimoga Rangappa, Padmalatha Kadirappa, Mallikarjun Adibatti, Pradeep Kumar Gowda

**Affiliations:** 1 Anatomy, Employees' State Insurance Corporation (ESIC) Medical College and Post Graduate Institute of Medical Sciences and Research (PGIMSR), Bengaluru, IND

**Keywords:** elbow joint, forensic anthropology, humerus length, linear regression analysis, prosthesis, radius, ulna

## Abstract

Introduction and aim

The distal end of the humerus consists of the trochlea and capitulum, which articulate with the ulna and radius to form the elbow joint. Fractures of the distal humerus are common and often require surgical fixation, while inflammatory arthropathies may necessitate elbow arthroplasty. Morphometric measurements of the humerus are useful in forensic anthropology for estimating humeral length and stature. Detailed knowledge of distal humeral morphology is also crucial for implant design and population-specific anatomical reference standards. This study aimed to determine the correlation between the total length of the humerus and its distal segmental dimensions, while emphasizing the morphometric significance of these segments in applied anatomy. To achieve this, the maximum length of the humerus, the epicondylar breadth, and maximum breadth of the articular surfaces at the distal end were measured and correlated with the total bone length.

Materials and methods

This study was conducted on 90 dry adult humeri (49 right and 41 left) with intact distal morphology obtained from the department of anatomy. Total humeral length was measured using an osteometric board. The maximum breadth of the distal end, as well as the individual widths of the capitulum and trochlea, were measured using a digital Vernier caliper. Descriptive tests, such as mean and standard deviation, along with inferential statistics, such as linear regression and the unpaired t-test, were used to derive the findings. Descriptive statistics and linear regression analysis were employed to evaluate the relationship between distal humeral dimensions and total humeral length.

Results

The mean maximum length of the right and left humeri was 273.23±43.57 mm and 262.95±46.49 mm, respectively. Linear regression analysis demonstrated a moderately positive relationship between total humeral length and all measured distal humeral segments. Regression equations were derived separately for right and left humeri, indicating that distal segment dimensions can reliably be used to estimate total humeral length.

Conclusion

Distal humeral measurements show a positive correlation with total humeral length and may aid in the estimation of stature when only fragmentary skeletal remains are available. These morphometric data may also contribute to the selection and design of prostheses for elbow replacement surgeries and provide population-specific anatomical reference values.

## Introduction

The humerus is the longest and largest bone of the upper limb. Its distal expanded end is a modified condyle that is transversely wide and consists of both articular and non-articular parts. The articular components include the lateral convex capitulum and the medial, pulley-shaped trochlea, which articulate with the radius and ulna, respectively. The non-articular parts comprise the medial and lateral epicondyles, along with the olecranon, coronoid, and radial fossae. The lower end of the humerus develops from several distinct ossification centers that mature at different times, with development typically occurring earlier in females. Key milestones include the capitulum (age one year), medial epicondyle (age four years), trochlea (age nine years), and lateral epicondyle (age 12 years), with the composite epiphysis uniting with the shaft around 14 years of age [1].

Skeletal remains of the humerus provide highly reliable data for biological profiling in contemporary populations and serve as a primary contributor to both biological sex estimation and living stature reconstruction. Morphometric analyses demonstrate marked sexual dimorphism, with males consistently exhibiting a greater maximum length, vertical head diameter, and epicondylar breadth than females. These humeral dimensions maintain a highly predictable, linear correlation with living height, enabling forensic anthropologists and bioarchaeologists to utilize mathematical regression equations to estimate total stature with high statistical accuracy. Even when skeletal assemblages are fragmented or incomplete, isolating specific segmental bony landmarks such as head diameter or shaft circumference provides alternative diagnostic metrics to extrapolate total humeral length and subsequently establish individual height [2,3].

However, because these morphological variations reflect the direct, lifelong influences of age, ancestry, environmental factors, nutritional status, and habitual occupational activities on bone remodeling, humeral dimensions are highly population-specific. Consequently, establishing population-specific osteometric standards is critical for accurate diagnostic application, while forensic practitioners must also account for the state of preservation, as fresh bones exhibit greater mean dimensions than dry specimens. The elbow joint is frequently involved in degenerative diseases like rheumatoid arthritis and complex fractures that cause permanent deformities. Accurate, population-specific anatomical metrics ensure that surgical treatments, such as open reduction, internal fixation, and prosthetic joint replacements, utilize implants that precisely match the patient's unique skeletal morphology. Consequently, establishing precise osteometric standards is critical not only for forensic diagnostics but also for optimizing orthopedic implant design and surgical outcomes [4-8].

## Materials and methods

Morphometric measurements were obtained from 90 dry adult humeri (49 right and 41 left) from separate individuals of varying ages and sexes, obtained from the bone repository of the Department of Anatomy, Employees' State Insurance Corporation (ESIC) Medical College and Hospital. To establish correlations and regression equations, we conducted an a priori power analysis using G*Power software version 3.1.9.7 (Düsseldorf, Germany: Heinrich-Heine-Universität Düsseldorf). Assuming a moderate expected correlation coefficient (effect size) of r=0.30, a significance level (α) of 0.05, and a standard power (1-β) of 0.80, the minimum required sample size was determined to be 84 specimens. Therefore, our actual sample size of 90 humeri is adequately powered to detect statistically significant relationships and ensures the validity of the planned regression analyses. Gender determination of the bone was not considered. Because no bilateral bones from the same individual were included in the sample, the assumption of statistical independence was fully satisfied, and no clustering adjustments were required for the analytical strategy. Damaged and deformed humeri were excluded from the study.

The maximum length of the humerus was determined using the osteometric board (Figures 1A-1D). Different segments in the distal end of the humerus were measured using a digital Vernier caliper. Activity on each parameter was repeated three times. Mean and standard deviation were applied to the values noted. All measurements were recorded in millimeters. Because the right and left bones belonged to different unknown individuals, an unpaired (independent) t-test was used to assess the significance of differences in length.

**Figure 1:**
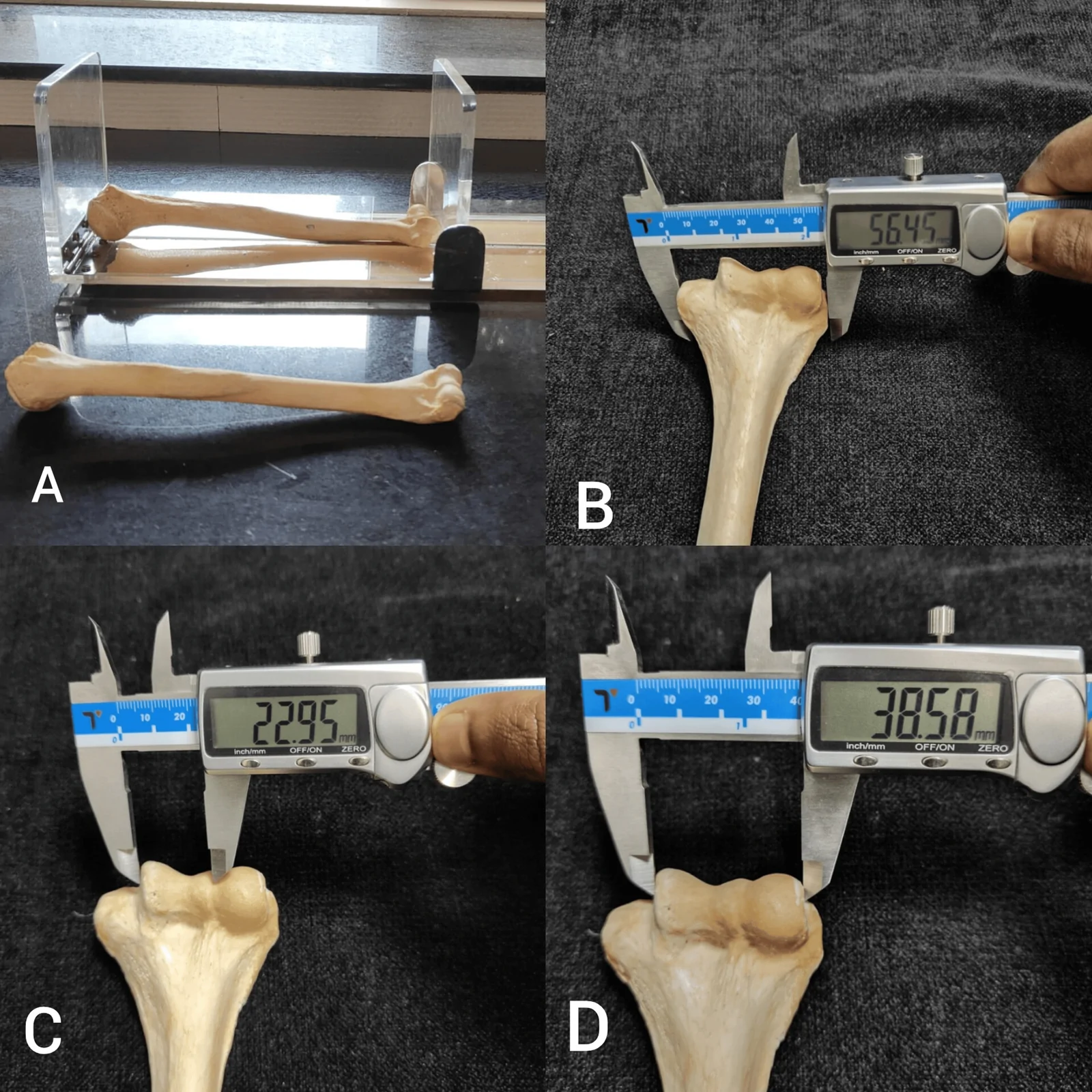
Images showing various measurements of humeri. (A) Measurement of the maximum length of the humerus using an osteometric board. (B) Measurement of the maximum distance between the medial and the lateral epicondyles. (C) Measurement of the maximum horizontal distance of the trochlea between the outer borders of the flanges. (D) Measurement of the maximum distance between the outer border of the medial flange of the trochlea and the lateral end of the capitulum.

The association between the distal segment and maximum length of the humerus was investigated by Pearson's correlation coefficient, and subsequently, linear regression was applied to analyze the right and left humeri independently. A probability (P) of <0.05 was considered statistically significant. All data analysis was done using Microsoft Excel (Redmond, WA: Microsoft Corp.) and Jamovi software version 2.7/2025 (Sydney, Australia: The jamovi Project Pty Ltd).

The following segments were measured from the humerus, and all measurements were documented in mm. M1 represented the maximum vertical length of the humerus, M2 represented the maximum distance between the lateral and medial epicondyles, M3 represented the maximum horizontal distance of the trochlea between the outer borders of both flanges, M4 represented the maximum distance between the medial flange of the trochlea and the lateral end of the capitulum, and M5 (M4-M3) represented the maximum breadth of the capitulum. The capitulum breadth was calculated indirectly via subtraction because its lateral border lacks a distinct, sharp anatomical landmark for direct caliper placement.

## Results

Morphometric measurements of the humeri are summarized in Table 1. The mean maximum humeral length (M1) was 273.23±43.57 mm for the right humeri and 262.95±46.49 mm for the left humeri. The mean maximum humeral length for both sides was 268.09±45.03 mm. The mean distal humeral breadth (M2) measured 56.87±4.03 mm on the right and 56.33±3.92 mm on the left. The mean trochlear width (M3) was 22.65±2.23 mm and 22.77±2.77 mm for the right and left sides, respectively. The mean distance between the medial flange of the trochlea and the lateral end of the capitulum (M4) was 39.98±3.42 mm on the right and 39.81±3.07 mm on the left. The mean capitular breadth (M5) was 17.29±2.32 mm on the right and 17.04±3.49 mm on the left. An unpaired t-test for the comparison of maximum length between the right and left humeri showed no statistically significant difference (p=0.0667, with alpha set at 0.05).

**Table 1:** Mean and standard deviation of right and left humerus segmental measurements. M1: maximum vertical length; M2: epicondylar breadth; M3: trochlear width; M4: total articular breadth from medial trochlear flange to lateral capitulum; M5: capitular breadth, calculated as M4-M3

Segments of the humerus	Right (n=49), mean±SD (mm)	Left (n=41), mean±SD (mm)
M1	273.23±43.57	262.95±46.49
M2	56.87±4.03	56.33±3.92
M3	22.65±2.23	22.77±2.77
M4	39.98±3.42	39.81±3.07
M5 (M4-M3)	17.33±4.65	17.04±3.49

Linear regression analysis was performed to evaluate the relationship between total humeral length (M1) and distal humeral measurements (M2, M3, M4, and M5). Table 2 summarizes the derived regression equations for the right and left humeri. The table indicates that M2 is the single best predictor of M1 on the right side, explaining 37.5% of its variance. On the left side, M4 is the best single predictor, explaining 27.2% of its variance. However, the derived variable M5 (M4-M3) holds no linear predictive value for M1 on either side; its p-values are extremely high (>0.50), and its R^2^ scores are near 0. In terms of consistency, both M2 and M4 remain highly dependable baseline anchors for predicting overall bone dimension (M1).

**Table 2:** Linear regression models for estimating total length (M1). M1: maximum vertical length; M2: epicondylar breadth; M3: trochlear width; M4: total articular breadth from medial trochlear flange to lateral capitulum; M5: capitular breadth, calculated as M4-M3

Side	Predictor variables	Linear regression equation	R^2 ^value	p-Value	Significance
Right	M2: epicondylar breadth	M1=7.74 (M2)-173.05	0.375	<0.0001	Highly significant
	M3: trochlear width	M1=6.44 (M3)-116.32	0.147	0.0153	Significant
	M4: total articular breadth	M1=7.40 (M4)-31.61	0.239	0.0012	Highly significant
	M5: capitular breadth (M4-M3)	M1=2.45 (M4-M3)+221.15	0.08	0.5895	Not significant
Left	M2: epicondylar breadth	M1=5.09 (M2)-16.03	0.221	0.0011	Highly significant
	M3: trochlear width	M1=7.37 (M3)+106.31	0.143	0.0113	Significant
	M4: total articular breadth	M1=6.63 (M4)+8.22	0.272	0.0001	Highly significant
	M5: capitular breadth (M4-M3)	M1=0.50 (M4-M3)+264.55	0.001	0.8037	Not significant

Pearson's correlation analysis demonstrated statistically significant positive correlations between total humeral length and all distal humeral parameters on both sides (Tables 3, 4). In the right humeri, moderate positive correlations were observed for M2, M3, and M4, while M5 showed a weak positive correlation. In the left humeri, M2 exhibited a strong positive correlation, whereas M3 and M5 demonstrated weak positive correlations. M4 showed a moderate positive correlation. All correlations were statistically significant (p<0.05). These findings indicate that distal humeral dimensions are positively associated with total humeral length, with distal humeral breadth (M2) showing the strongest predictive relationship, particularly on the left side.

**Table 3:** Pearson's correlation coefficients (r) and significance levels between total humeral length (M1) and distal humeral parameters (M2-M5) in right humeri (n=49). *Correlation is significant at the 0.01 level (2-tailed). **Correlation is significant at the 0.05 level (2-tailed). M2: epicondylar breadth; M3: trochlear width; M4: total articular breadth from medial trochlear flange to lateral capitulum; M5: capitular breadth, calculated as M4-M3

Distal humeral parameters	Correlation coefficient (r)	p-Value	Strength of relationship
M2: epicondylar breadth	0.4569	0.0010*	Moderate positive linear
M3: trochlear width	0.4447	0.0014*	Moderate positive linear
M4: total articular breadth	0.5214	<0.001*	Moderate positive linear
M5: capitular breadth (M4-M3)	0.3438	0.0156**	Weak positive linear

**Table 4:** Pearson's correlation coefficients (r) and significance levels between total humeral length (M1) and distal humeral parameters (M2-M5) in left humeri (n=41). *Correlation is significant at the 0.01 level (2-tailed). **Correlation is significant at the 0.05 level (2-tailed). M2: epicondylar breadth; M3: trochlear width; M4: total articular breadth from medial trochlear flange to lateral capitulum; M5: capitular breadth, calculated as M4-M3

Distal humeral parameters	Correlation coefficient (r)	p-Value	Strength of relationship
M2: epicondylar breadth	0.6151	<0.001*	Strong positive linear
M3: trochlear width	0.3725	0.0012**	Moderate positive linear
M4: total articular breadth	0.4885	0.0165*	Weak moderate positive linear
M5: capitular breadth (M4-M3)	0.3113	0.0476**	Weak positive linear

## Discussion

This morphometric analysis reveals that all p-values are greater than 0.05; there is no statistically significant difference in any of the structural measurements between the right and left samples. The distal humeral parameters, particularly distal breadth (M2), possess a strong predictive relationship with overall humeral length. Epicondylar breadth (M2) exhibited a high predictive value on the left side (r=0.6151), whereas total articular breadth (M4) proved to be more effective on the right side (r=0.5214). Mechanistically, these differences may be attributed to directional asymmetry resulting from lifetime biomechanical strains, as well as confounding variables such as the age and sex distribution within the sample population.

The length of the humerus is useful in estimating the average height of the individual, an important aspect in identification. This provides critical data for forensic identification. The studies have also confirmed the difference in various population groups. Turkish population has a significantly longer humerus. This has been confirmed by studies conducted on living individuals [9].

The mean value of total length is evidence to indicate the characteristic feature of a population as a whole. Individual stature can be estimated using a linear regression formula. The regression analysis and the formulas obtained in the present study correlate with similar studies conducted earlier [10]. Across both cohorts, the derived regression models show that even when long bones are highly fragmented, isolated distal landmarks such as the epicondyles and segments of the trochlear-capitular complex can effectively support stature and skeletal identification workflows.

However, our findings underscore the necessity of applying population-specific standards in anthropological analysis. Comparative morphometric data reveal notable discrepancies when standard formulas are applied indiscriminately across varying ancestries. Specifically, population groups such as the Turkish adult cohort have been shown to possess significantly longer humeral mean dimensions compared to various global reference populations. This emphasizes that genetic, environmental, and regional factors heavily influence skeletal proportions.

A chronological review of comparative morphometric literature demonstrates that the parameters observed in the present study align well with established global baseline distributions, while highlighting distinct regional variations (Table 5). The pooled mean maximum length of the humerus in our cohort (268.09±45.03 mm) falls within the broad historical spectrum of 259.91 mm reported by Agarwal and Gopal and the upper bound of 308.38 mm documented in the Turkish adult population by Mutluay et al. [11,12]. Notably, our sample exhibits a slightly shorter mean length compared to the majority of recent studies, which consistently hover above 300 mm (e.g., Havaldar et al.; Azhagiri et al.), underscoring the vital importance of population-specific and localized reference lines [13,14].

**Table 5:** Chronological review of literature comparing bilateral humeral parameters. All values are presented in mm.

Studies	Sample size (n)	Maximum length (M1), mean±SD	Epicondylar distance (M2), mean±SD	Trochlear width (M3), mean±SD	Capitular breadth (M5), mean±SD
Present study (2026)	90	268.09±45.03	56.60±3.99	22.71±2.44	17.12±2.06
Sailaja et al. (2026) [8]	100	-	56.10±2.0	-	-
Havaldar et al. (2025) [13]	100	305.0±2.26	58.50±7.0	-	-
Azhagiri et al. (2024) [14]	100	306.69±22.99	57.23±4.25	-	-
Agarwal and Gopal (2023) [11]	115	259.91±2.50	-	22.50±2.89	15.66±1.77
Vinay et al. (2021) [15]	200	303.73±22.21	56.72±4.76	-	-
Afzal et al. (2024) [16]	70	304.50±13.75	58.52±3.01	22.97±1.75	19.40±0.45
Prasad et al. (2017) [17]	200	299.76±22.60	-	-	-
Khan et al. (2020) [7]	174	290.58±10.89	56.98±2.90	-	-
Sinha et al. (2020) [18]	60	307.58±8.46	-	-	-
Mutluay et al. (2020) [12]	100	308.38±21.02	-	-	-
Somesh et al. (2011) [10]	100	304.60±21.55	-	-	-

In contrast to the highly variable long bone lengths, the distal extremity parameters demonstrate remarkable morphological stability across cohorts. The mean maximum epicondylar distance (56.60±3.99 mm) closely mirrors the findings of Sailaja et al. (56.10±2.00 mm) and Vinay et al. (56.72±4.76 mm) [8,15]. Similarly, the mean trochlear width (22.71±2.44 mm) and capitular breadth (17.12±2.06 mm) show strong statistical continuity with the values reported by Afzal et al. [16]. This marked structural consistency across geographically diverse timelines reinforces the clinical utility of the distal humerus as a reliable, stable metric template for surgical implant engineering and forensic reconstruction [18].

Limitations

While this study provides valuable anatomical data for humeral sizing and implant design, several limitations must be acknowledged. First, the use of dry bones means the exact age, sex, stature, and nutritional history of the individuals were unknown, which prevents analysis of demographic variations. Second, the absence of overlying articular cartilage and soft tissue may slightly alter the precise measurements compared to living subjects. Third, the sample size of 90 humeri was sourced from a specific regional population, meaning the morphological correlations may not fully represent other geographic or ethnic groups. Finally, despite careful manual measurements, minor human error and intraobserver variability in pinpointing anatomical landmarks cannot be completely ruled out.

## Conclusions

The present morphometric study confirms that specific fragments of the human humerus, particularly its distal parameters, are highly reliable and mathematically robust skeletal indicators for indirectly reconstructing total bone length, reinforcing their diagnostic utility in forensic and bioarchaeological applications. While the total length of the bone (M1) exhibits significant regional variation that underscores the critical necessity of population-specific reference models, the intra-articular and distal parameters (M2 through M5) display remarkable morphological stability. Furthermore, the strong bilateral symmetry (p>0.05) demonstrated between the right and left distal metrics establishes the validity of using the contralateral limb as a dependable diagnostic or reconstructive template, ultimately enhancing our understanding of the functional anatomy of the elbow joint.
